# The reference-independence of CSR expectations for luxury firms

**DOI:** 10.1371/journal.pone.0287014

**Published:** 2023-06-15

**Authors:** Jared Wong, Foo Nin Ho

**Affiliations:** 1 Department of Marketing, Yale School of Management, New Haven, Connecticut, United States of America; 2 Department of Marketing, Lam Family College of Business, San Francisco State University, San Francisco, California, United States of America; Yunnan Technology and Business University, CHINA

## Abstract

Consumers actively look to and expect businesses to engage in charitable donation activities. While past research has demonstrated the strategic benefits that corporate social responsibility (CSR) affords to firms, little is known about the way consumers apply subjective (or objective) ethical standards for corporate donations. Our research focuses on the way expectation standards of CSR are applied to luxury (versus non-luxury) companies. Do consumers hold a belief that luxury firms are expected to donate more? Four experimental studies find robust and converging evidence that consumers do not hold luxury firms to a higher standard; instead, they take on the normative belief that companies are obligated to donate equal amounts. This reference-independence holds stable across different product categories (Studies 1a and 1b), perspectives (Study 2), and attempts to alter the belief (Study 3). However, individual differences do exist among consumers regarding the level of donation expected, particularly for materialists and spendthrifts. Specifically, moderation analyses reveal that materialists and spendthrifts (compared to non-materialists and tightwads) expect higher levels of corporate donations regardless of the type of firm (i.e., luxury vs. non-luxury). This research extends the discussion of subjective ethical beliefs in the context of luxury CSR.

## Introduction

During the COVID-19 pandemic, the public’s knowledge about corporate donations became especially salient, as consumers held expectations that companies make donations to aid in the time of crisis [1–3]. For example, in March of 2020, luxury conglomerate LVMH quickly instituted a series of corporate social responsibility (CSR) initiatives, including the production of essential materials, donations to relief organizations, and the distribution of face masks [4] [cf. price gouging of essential goods during the pandemic [5]]. Inspired by the way luxury firms participated in CSR activities during the COVID-19 pandemic, we ask the following research question: Do consumers assign higher donation obligations to luxury (compared to non-luxury) firms? Extant research has examined the important role that CSR plays in the minds of consumers. As demanded by consumers, the growth of CSR [6], or a firm’s obligation and responsibility to society [7], other than its stockholders and beyond prescribed by law [8, 9] has become increasingly observable. In addition, research has pointed to the ways CSR provides strategic benefits for a firm [10, 11], ranging from tax deductions [12] to brand awareness [13] and economic and reputational benefits [14, 15].

Given the expectation that consumers hold for firms to make a positive contribution to society, our research unearths a research question previously unanswered: Does the luxuriousness of a firm influence the extent to which consumers hold donation expectations? Consideration of this question involves conceptualizing normative versus descriptive beliefs [16] about the ethics of donation obligations. One normative model of judgment may suggest that all companies, regardless of what is sold, should strive to contribute to society; therefore, consumers would expect commensurate levels of donations among all firms. This model of judgment describes a *reference-independent* orientation; that is, a judgment is made without alteration to what or whom the judgment is being made about. Another normative model can argue that firms with the highest profits should be the ones with the highest donation obligations, as companies with higher profit sacrifice less when making a donation. A third model involves associating firms that produce durable, long-lasting goods with having a positive impact on the environment, as consumers do not have to repeat purchases for the same product. If true, recent research indicates that luxury firms would actually be associated with these benefits [17]. All three modes approaches presuppose that consumers do not hold higher subjective standards for a luxury firm’s CSR activities.

On the other hand, descriptive models of judgment may suggest that as luxury firms are associated with excess and wealth, consumers may impose higher standards for luxury firms to donate. This orientation is *reference-dependent*, whereby a judgment is made dependent upon the object, subject, or person that the judgment concerns. It can be conceived that non-luxury firms do not have these associations, and consumers expect these firms to donate less. On the other hand, consumers may perceive luxury firms to be less genuine when making donations, and thus, expect “compensatory donations.” This compensatory donation obligation may be salient as consumers are suspicious about a luxury firm’s true donation intentions and thus expect higher donation obligations from luxury firms.

Overall, our research considers ethical donation obligation judgments as a reference-dependent or reference-independent phenomenon. Do consumers believe luxury firms or non-luxury firms should donate more? Or, is it the case that consumers impose equivalent standards of donation expectations across all firms, irrespective of whether the firm sells luxury products?

This article attempts to address the growing need for marketing scholars to consider transformative luxury research [18]. We depart from traditional luxury research that focuses on consumer purchasing decisions, and, instead, consider the way consumers impose donation obligations onto luxury firms. Transformative luxury research has the potential to elaborate upon the role that luxury firms play in the marketplace, and one important consideration concerns CSR. Our research also aligns with recent research that has demonstrated the way consumers have subjective ethical giving standards. For example, Berman, Bhattacharjee [19] explore how an individual’s income, in reference to a social other’s, drives subjective standards of generosity. Drawing from the evidence that consumers “pass the buck” in terms of donation obligations to higher-earning others, who also pass the buck to even wealthier individuals, our research elaborates upon this “passing the buck” effect by exploring if reference-dependence also generalizes to corporations.

## Subjective ethics for luxury CSR and its potential reasons

A stakeholder’s attribution for CSR initiatives can be conceptualized as extrinsic (i.e., consumers view the initiative as trying to grow profit) or intrinsic (i.e., the initiative stems from a real concern for the focal issue) [20]. As Ginder, Kwon [21] note, “[i]n addition to the use of CSR communication as a means for positive impression management, *CSR*-*washing*, or the phenomenon where a company presents itself as being more socially responsible than it actually is, has intensified the level of wariness some consumers have toward CSR publicity.” Research has discussed how a luxury brand’s self-enhancement concept is in conflict with the CSR information’s self-transcendence concept [22]; this mismatch between luxury and CSR has led researchers to argue that CSR activities for luxury firms are an ineffective strategy. Others have called this a “Catch-22 of responsible luxury,” where the terms “responsible” and “luxury” are at odds with one other [23]. Scholars have even asked the question, “Can luxury brands even be ethical” [24]? From the managerial perspective, the perception of the low environmental impact of the luxury industry leads some luxury brand managers to be reluctant to even embrace CSR [25].

In the present article, we build upon recent luxury research that claims that luxury consumption is a more sustainable approach for consumers [17]. Consistent with this thinking, and reflecting recent trends whereby luxury conglomerates are making donations (such as in the example explored in the Introduction), luxury firms have been engaging in (and embracing) CSR activities. The collection of research that argues “luxury” and “CSR” are mutually exclusive says little about the implications for such a contradiction. Our research advances the literature on this topic by examining the way consumers make objective donation assignments in light of this supposed contradiction. Interest in the intersection of business ethics and luxury brands has been growing; for example, Septianto, Kemper [26] explored how pride and gratitude appeals in advertising play a role in an individual’s eWOM about sustainable luxury brands. Others have examined how social media influencers reconcile ethicality with living a luxurious life [27], or how nationalistic appeals can actually spur greater interest in foreign luxury brands [28].

Next, consider the example by Berman, Bhattacharjee [19]; their research indicates that less wealthy individuals see wealthier individuals as having a higher donation obligation because higher incomes are associated with more financial slack (i.e., an abundance of spare money) [29]. These subjective giving standards arise from judgments about higher earners’ spare money. What underlies these evaluations include an overestimation of the spare financial resources of higher earners. Our research considers a related but slightly different context. We consider if consumers perceive luxury firms in the same way that they judge wealthier social others, as luxury and wealth are interconnected concepts. However, the difference arises as both luxury and non-luxury firms can be “wealthy” (i.e., have high profits). Yet, this lay intuition in consumer’s minds about the connection between luxury and wealth may obfuscate that non-luxury firms have the potential to be much larger companies. Therefore, unlike a wealthier individual, the luxury firm may actually have less leeway to make donations to charitable causes, yet consumers may still expect them to do so.

What the present research explores is if consumers also engage in subjective ethical judgments for corporate donation activities. Research has indicated that displays of conspicuous consumption are interpreted by others as morally objectionable [30], yet both inconspicuous and conspicuous luxury signals still are expensive, exclusionary, and used as status signals [31–33]. Even so, it is inconspicuous brands that are seen as more morally acceptable [30]. Given that luxury products can be seen as morally objectionable, excessive [34], and resultant of social costs to the consumer [35], consumers may make a number of judgments regarding donation obligations: First, individuals may be skeptical about the genuineness of a luxury firm’s CSR activities; second and similarly, they may believe these charitable activities do not stem from a sincere desire to help others; and third, consumers may believe that luxury products are superfluous items, and they could transfer this belief onto luxury firms.

Attitude towards a company’s social responsibility involves how genuinely an individual believes an organization cares about a focal charitable cause [36]. As research indicates, CSR is becoming an *ambiguous* signal of organizational goodwill, whereby companies may be seen as engaging in donations out of self-interest. Further complicating this ambiguous signal includes media depictions of questionable CEO ethical behavior, which contradict their company’s CSR motives [37]. From a stakeholder perspective, investors are able to distinguish between genuine CSR efforts and “cheap talk” [38].

In this present research, we also include potential moderators—traits relating to spendthrifts and materialists—that may play a role in this process. For instance, the spendthrift-tightwad construct measures if a consumer has a tendency to experience psychological discomfort when making or anticipating a purchase [39]; materialism refers to the belief that goods are a means to happiness—that life satisfaction stems from possession and consumption [40]. It can be hypothesized that spendthrifts and materialists do not morally object to luxury goods—instead they may embrace luxury consumption. Research has drawn a direct link between a consumer’s level of materialism and their desire to make luxury purchases [41–43]. Similar connections have been made between spendthrifts and luxury consumption [44, 45].

## Overview of studies

Our research goal is to uncover if consumers make reference-dependent (i.e., subjective) or reference-independent (i.e., objective) judgments about corporate giving. While research has indicated that reference-dependence operates in social interactions, little is known about the judgments made by consumers about a company’s CSR activities based on the product the company sells. As delineated, there are two opposing arguments that can be made. First, consumers may hold the normative belief that all companies should be held to a high standard of giving. If true, the following studies would indicate that there exists no significant difference between the judgments made for luxury and non-luxury firms. Furthermore, if this belief is robust and stable, an attempt to highlight the negative [positive] societal impacts of luxury firms would not alter the pattern of results. Finally, even if participants are asked to switch their perspective (i.e., from a consumer to a manager), a stable belief that donations should be objective, and reference-independence would prevail.

On the other hand, a belief that luxury firms should be held at a higher standard would reveal a different pattern of results. If this line of thinking holds true, the following results would indicate that consumers hold higher expectations for a luxury firm’s corporate giving. As such, consumers apply compensatory expectations on luxury firms in the form of higher donation standards. Our research therefore is left as an empirical question, where the following four studies attempt to discriminate between these two predictions.

Four experimental studies demonstrate that consumers hold a stable and objective standard for the CSR activities of both luxury and non-luxury firms. Across two distinct product categories, Studies 1a and 1b explore the focal effect that consumers do not pass on higher obligations to luxury firms when the firm is making a donation decision. Study 2 considers how consumers apply objective or subjective donation standards when the perspective is switched, that is, when they imagine themselves to be a manager making a CSR decision. In Study 3, we employ experimental stimuli meant to override the belief in reference-independence but find that this conviction is stable.

An *a priori* power analysis was conducted before every study to ensure a sufficient number of participants to detect a medium effect size. All power analyses were set to detect 80% power at a squared multiple correlation ρ^2^ of 0.6. Actual participant numbers sampled exceed the suggested minimum by the power analyses in order to be as conservative as possible. All data can be found on our Open Science Framework page (https://osf.io/tfbjd/).

### Ethics statement

For all studies, informed verbal consent was obtained. The studies were given an exemption from the Institutional Review Board at San Francisco State University.

## Study 1 (1a and 1b): Establishing objective donation standards

### Overview and method

Study 1a uses the context of automobiles to examine the potential differential (or equivalent) way consumers assign donation obligations to luxury and non-luxury firms; Study 1b considers the context of department store companies. Both studies employ a one-way experimental design, whereby the stimuli (a luxury vs. non-luxury brand) is randomly assigned. For example, in Study 1a, we use Mercedes-Benz to represent a luxury automobile manufacturer, while Volkswagen is used to represent the non-luxury counterpart. The brands were chosen on the basis that they varied on luxuriousness; likewise, both are German companies, as we wanted to keep the country constant to reduce potential confounding from country-of-origin effects, especially for conspicuous and/or luxury goods [46, 47]. To ensure that consumers were able to distinguish between the two brands, a pre-test was run with 103 Amazon Mechanical Turk (MTurk) participants who answered the following question: “To what extent do you think the following brand represents a luxury brand?” (1 = *not at all*, 7 = *very much*). In the pre-test, respondents were asked to report their perceived luxuriousness among the automobile companies Audi, Mercedes-Benz, Porsche, Volkswagen. They were also given the option to state that they could not determine the luxuriousness of the brand (i.e., they were unfamiliar). Of the list of brands, about 10% were not able to make a judgment about Porsche nor Audi. By and large, consumers felt the most confident about making an evaluation for Mercedes-Benz and Volkswagen. As predicted, the pre-test confirmed that consumers perceive Mercedes-Benz to be more luxurious than Volkswagen, t(193) = 3.831, p < .001.

In addition to generalizing the findings to another product category, Study 1b serves as a conceptual replication of the results in Study 1a. In Study 1b, we use the context of clothing department stores. Target was selected to represent a non-luxury store, and Nordstrom a luxury store. The two brands were selected in order to hold the country-of-origin (i.e., United States) constant. The pre-test ensured that consumers were able to identify that these companies differ in terms of luxuriousness. Pre-test participants were given a list of department store brands, including Target, Macy’s, Nordstrom, and Neiman Marcus. Respondents were least familiar with Neiman Marcus and Macy’s and felt most confident about judging the luxuriousness of Target and Nordstrom. As expected, Nordstrom was rated to be more luxurious than Target, t(175) = 2.361, p = .019.

We drew from an MTurk sample of 262 participants (66% male; Mo_Age_ = 25–34; Mo_Education_ = Bachelor’s Degree) for Study 1a and 253 participants (68% male; Mo_Age_ = 25–34; Mo_Education_ = Bachelor’s Degree) for Study 1b. Similar to the method employed by Berman, Bhattacharjee [19], all participants read the following scenario: “The automobile company Mercedes-Benz [Volkswagen] is looking to donate a percentage of its profits to a charity.” Then, participants’ expectation for donations were measured both quantitatively and qualitatively. The former approach asks the participant, “What is the appropriate percentage of profit that this company should donate to the charity? This number should reflect what you think is morally appropriate.” Respondents reported a percentage between 0% and 100%. On a seven-point scale, the latter dependent measure asks the question, “To what extent do you believe that this company has a moral obligation to donate a percentage of its profit to a charitable cause?”.

Finally, the spendthrift-tightwad scale was adapted from Rick, Cryder [39] [Study 1a: r(218) = .574, p < .001; Study 1b: r(217) = .533, p < .001] and the materialism scale (Study 1a: α = .730; Study 1b: α = .743) from Richins [48]. A short-form version of original spendthrift-tightwad scale was used. These items included an 11-point semantic differential scale and a description scored on a five-point scale. For the materialism scale, one reversed-scored item, “People place too much emphasis on material things” was excluded for having poor reliability in our analysis. All other items were retained.

In both Studies 1a and 1b, participants were randomly assigned to the luxury or non-luxury conditions. All measurement scales in Study 1a were also used in Study 1b. The presentation of the scales was counterbalanced to prevent order effects (Haugtvedt and Wegener 1994); an attention check was included in both Study 1a and 1b that asked the participant which brand (i.e., Mercedes-Benz [Nordstrom] vs. Volkswagen [Target]) was included in the scenario that they read. After excluding the responses that failed the attention check, 220 remained for Study 1a, and 219 remained for Study 1b.

### Study 1a: Results

#### Quantitative donation expectations

With the quantitative donation expectation measurement as the dependent variable, consumers had no significant evaluation differences between luxury (M = 54.36, SD = 29.45) and non-luxury firms’ donation obligations (M = 55.08, SD = 25.97), t(218) = .186, p>.05, d = .025. We then test for moderation with Hayes’ PROCESS Model 1 [49] with the moderating variable of spendthrift-tightwad, F(3, 216) = 36.715, p < .001, R^2^ = .338. There was significant main effect for the spendthrift-tightwad variable on quantitative donation expectations, b = 10.947, t(216) = 9.815, p < .001. That is, controlling for the type of company, consumers who scored higher on the spendthrift scale (i.e., those who do not experience psychological discomfort when making a purchase) tend to expect higher levels of donations from companies. When testing for potential moderation from materialism, we do find a significant overall model, F(3, 216) = 5.621, p = .001, R^2^ = .072. The interaction was not significant, b = 3.448, t(216) = 1.579, p>.05; however, there was a main effect from materialism, b = 8.640, t(216) = 3.957, p < .001. That is, consumers who scored higher on materialism (i.e., those who hold the belief that consumption is a means to happiness) had significantly higher expectations for corporate donations.

#### Qualitative donation expectations

**Fig 1:**
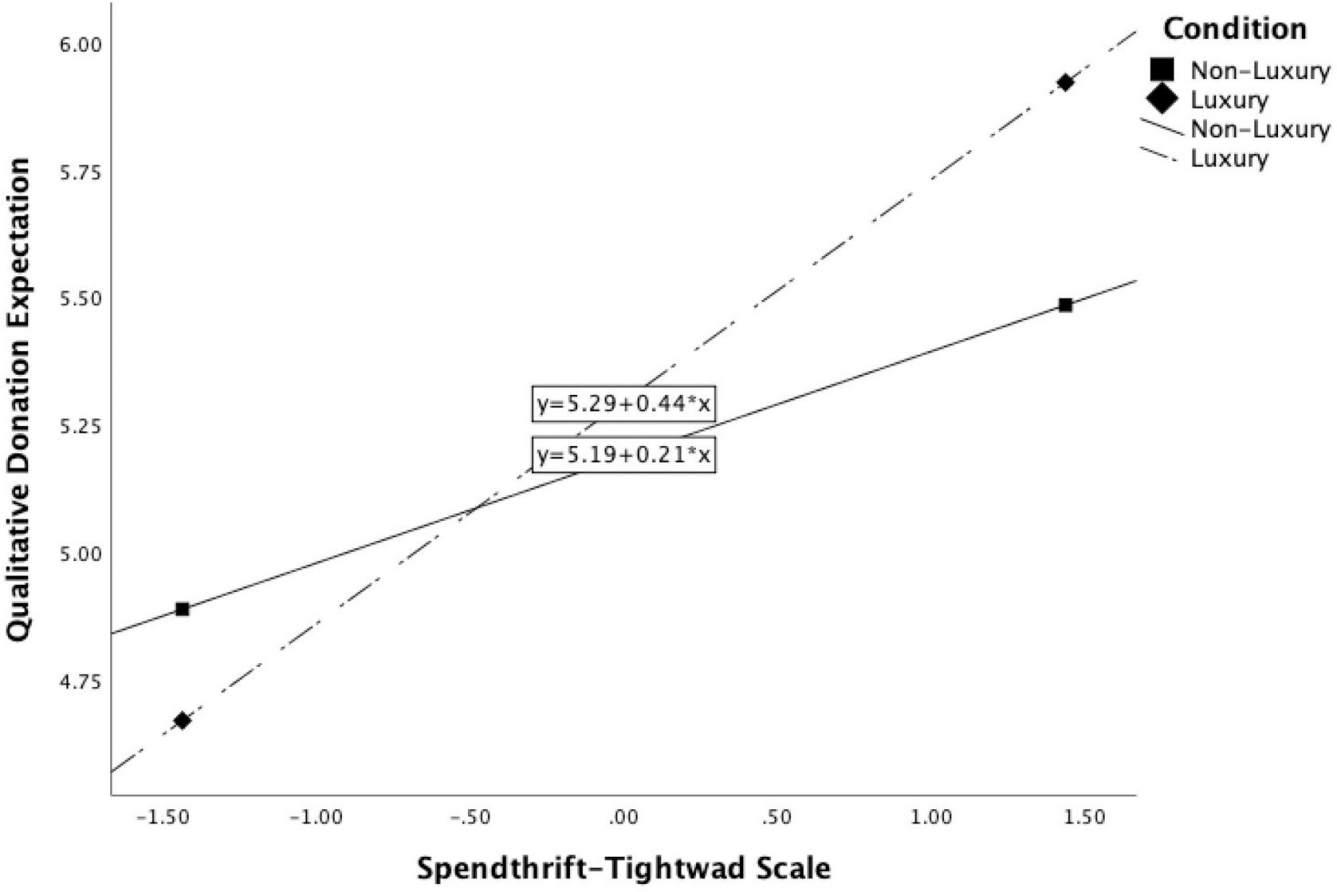
Interaction between the luxury condition and the spendthrift-tightwad construct.

The same set of statistical tests were applied with the dependent variable of qualitative donation expectations. Like the quantitative donation expectations, the qualitative evaluation also did not differ dependent upon judging a luxury (M = 5.30, SD = 1.31) or non-luxury firm (M = 5.18, SD = 1.15), t(218) = .687, p>.05, d = .094. The overall moderation model for spendthrift-tightwad was significant, F(3, 216) = 16.152, p < .001, R^2^ = .183. The interaction between the condition (luxury vs. non-luxury) and the spendthrift-tightwad variable was significant, b = .114, t(216) = 2.077, p < .05; likewise, the main effect of the spendthrift-tightwad variable was also significant, b = .322, t(216) = 5.858, p < .001. The addition of the interaction was a significant change to the model, F(1, 216) = 4.315, p < .05, ΔR^2^ = .016. For spendthrift values [1 SD (i.e., 1.436) below the mean], the condition of the firm does not predict the qualitative expectation of corporate giving, b = -.109, t(216) = -.992, p>.05. At the average level of scores on the spendthrift-tightwad scale (i.e., at the mean), the condition of the firm also does not predict the qualitative assessment of donations, b = .055, t(216) = .709, p>.05. For high spendthrift scores (1 SD above the mean), whether the firm sold luxury or non-luxury products did predict the expectation of donations such that spendthrift consumers do hold higher standards of giving for luxury firms, b = .219, t(216) = 1.978, p < .05. The Johnson-Neyman significance region begins when a consumer scores 1.415 above the spendthrift-tightwad mean (see Fig 1).

**Fig 2:**
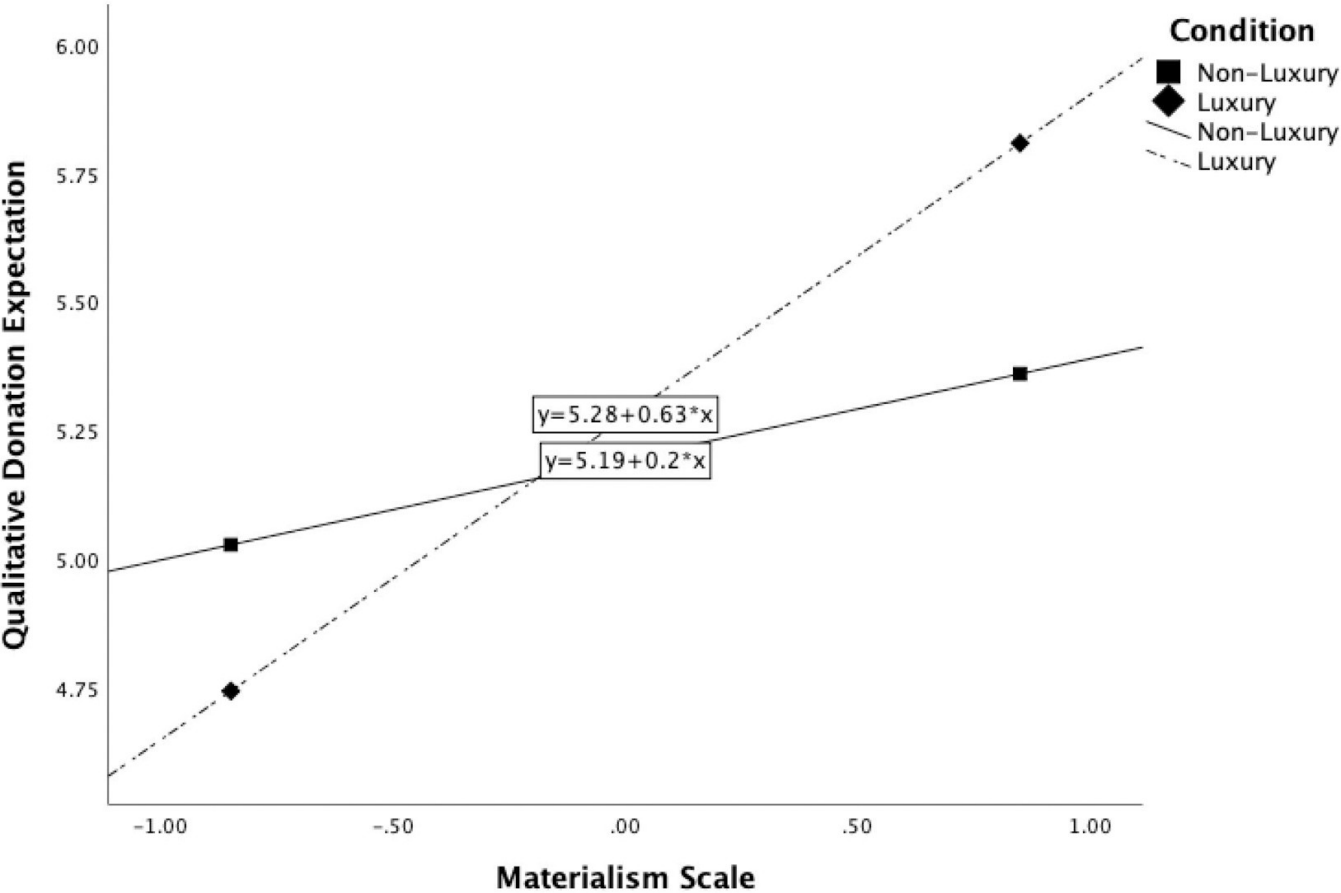
Interaction between the luxury condition and the materialism construct.

Next, another moderation analysis was run with the materialism variable, finding a significant overall model, F(3, 216) = 7.324, p < .001, R^2^ = .092. Both the main effect of materialism, b = .412, t(216) = 4.302, p < .001, and the interaction effect between materialism and the luxury [non-luxury] condition were significant, b = .216, t(216) = 2.256, p < .05. Therefore, as the materialism score increases, the higher the consumer’s expectations for donations are. The addition of this interaction was a significant change to the model, F(1, 216) = 5.089, p < .05, ΔR^2^ = .021. All conditional effects below, b = -.041, t(216) = -1.238, p>.05, and above 1 standard deviation (i.e., .848) from the mean, b = .224, t(216) = 1.950, p>.05, in addition to at the mean, b = .041 t(216) = .505, p>.05, were not significant. The Johnson-Neyman significance region begins when the materialism score is .878 above the mean (see Fig 2).

### Study 1b: Results

#### Quantitative donation expectations

Participants revealed no significant evaluation difference between luxury (M = 53.50, SD = 29.08) and non-luxury firm’s donation obligations (M = 56.40, SD = 27.17), t(217) = .762, p>.05, d = .103. When testing for moderation from the spendthrift-tightwad variable, we find a significant overall model, F(3, 215) = 24.534, p < .001, R^2^ = .255. Replicating the results from Study 1a, there was no main effect for the condition (luxury vs. non-luxury), nor was there an interaction between the condition and spendthrift-tightwad, b = 2.446, t(215) = 1.248, p>.05. Likewise, there was significant main effect for the spendthrift-tightwad variable on quantitative donation expectations, b = 15.611, t(215) = 7.901, p < .001. That is, spendthrifts expect higher levels of donations from companies. When testing for potential moderation from materialism, we find a significant overall model, F(3, 215) = 4.081, p < .01, R^2^ = .054. The interaction was not significant, b = 2.315, t(215) = 1.051, p>.05, however there was a main effect from materialism, b = 6.642, t(215) = 3.016, p < .01. Therefore, materialistic consumers had significantly higher expectations for corporate donations.

### Qualitative donation expectations

No differences were detected between the evaluation of a luxury (M = 5.23, SD = 1.26) or non-luxury firm (M = 5.12, SD = 1.44), t(217) = .624, p>.05, d = .084. The overall moderation model for spendthrift-tightwad was significant, F(3, 215) = 9.120, p < .001, R^2^ = .113. The interaction between the condition (luxury vs. non-luxury) and the spendthrift-tightwad variable was not significant, b = .045, t(215) = .437, p>.05; however, the main effect of the spendthrift-tightwad variable was significant, b = .509, t(215) = 4.917, p < .001. Next, another moderation analysis was run with the materialism variable, finding a significant overall model, F(3, 215) = 7.995, p < .001, R^2^ = .100. The main effect of materialism was significant, b = .493, t(215) = 4.783, p < .001; the interaction effect between materialism and the condition (luxury vs. non-luxury) was not, b = -.169, t(215) = -1.637, p>.05.

## Discussion

Study 1a brings to light two important findings regarding the subjectivity of ethical CSR evaluations. First, unlike in the social context, consumers do not hold reference-dependent standards for ethical corporate donations; instead, consumers hold reference-independent beliefs. Regardless of the reference firm (i.e., luxury vs. non-luxury), consumers do not believe that one type of firm has a higher donation obligation over another. Instead, both luxury and non-luxury firms are held to the same standard. However, moderation analysis that examines individual differences gives more nuanced findings. Primarily, a consumer’s level on the spendthrift-tightwad and materialism scales are associated with the level of donation expectation. More specifically, consumers who are spendthrifts and materialists expect higher levels of donations from companies. Furthermore, Study 1b provides a successful conceptual replication of the pattern of results found in Study 1a. Study 1b’s results replicate that consumers hold reference-independent beliefs concerning CSR activities and that individual differences play an important role. Consequently, by changing the product category in Study 1b, we find that the results can generalize (i.e., are not confined to the product category chosen in Study 1a).

## Study 2: Switching the perspective

### Overview and methods

Studies 1a and 1b demonstrate that consumers hold reference-independent beliefs when making evaluations about a company’s donation expectations. Study 2 considers a switched perspective: How do consumers apply objective or subjective donation standards when they imagine themselves as a manager making a CSR decision [i.e., the normative prescriptions of managerial behavior and the role of the chief executive [50–52]]? While Studies 1a and 1b examine this question from the perspective of a consumer holding donation expectations for a company, Study 2 explores if consumers imagining themselves as the ones making the decision changes the way they apply differential judgments onto companies. Randomly assigned to the luxury or non-luxury conditions, participants read the following prompt: “Imagine you are a manager for the automobile company Mercedes-Benz [Volkswagen], and the company wishes to make a donation to a charitable cause.” Then, respondents were presented with the qualitative measurement for donation obligation, which asked the participants on a seven-point, Likert scale the following question: “To what extent do you believe that this company has a moral obligation to donate a percentage of its profit to a charitable cause?” The questionnaire concluded with an attention check, ensuring that the participants could report which brand was shown to them. Two hundred and forty-nine participants were recruited from MTurk (69% male; Mo_Age_ = 25–34). Thirty-five participants were excluded for failing the attention check.

## Results

An analysis revealed that there still does not exist any donation expectation differences between luxury (M = 5.53, SD = 1.23) and non-luxury firms (M = 5.23, SD = 1.33), t(211) = 1.722, p>.05, d = .237.

### Discussion

Our results indicate that when asked to take on a managerial perspective, participants still hold reference-independent beliefs. Thus, Study 2 continues to lend support to the pattern of results found in Studies 1a and 1b. Specifically, Study 2 indicates that even with a switched perspective, individuals still believe that both luxury and non-luxury firms have similar obligation standards.

### Study 3: The stability of the reference-independent belief

#### Overview and methods

The results from Studies 1a, 1b, and 2 demonstrate that consumers do not transfer a tax on luxury firms in the form of higher donation obligations. Study 3 explores how stable this reference-independent belief is (i.e., how easily it can be altered). As Sun, Bellezza [17] note, consumers often fail to consider the positive environmental impacts from purchasing fewer but higher-end products. In Study 3, consumers were tasked with reading a passage that explains how luxury firms pay workers more [less] fairly and contribute less [more] to environmental pollution. This stimulus was designed as a method to potentially alter a consumer’s belief that luxury firms should (or should not) be held to a higher standard than non-luxury firms. A separate pre-test (n = 103) confirmed that consumers associate fair wages and a reduction in environmental pollution with a moral company (α = .631). Four hundred MTurk participants (72% male; Mo_Age_ = 25–34) were randomly assigned to a 2 (luxury companies are moral vs. immoral conditions) x 2 (luxury vs. non-luxury brand conditions) experimental design. Forty-eight failed the manipulation check and were excluded from the analysis. Qualitative and quantitative assessments of donation obligation expectations were measured. Given that Studies 1a, 1b, and 2 converge on the conclusion that individuals hold reference-independent beliefs about donation expectations, should this belief be stable (i.e., not easily subject to change even given disconfirming evidence), then we would expect no significant differences between the expectations given to luxury and non-luxury firms.

## Results

For quantitative donation expectations, neither the moral condition, F(1, 348) = .114, p>.05, nor the luxury condition, F(1, 348) = .082, p>.05, had significant main effects. See Table 1 for the results. Furthermore the interaction was also non-significant, F(1, 348) = .082, p>.05. Likewise, with qualitative donation expectation as the dependent variable, we find that the moral condition did not have a significant main effect, F(1, 348) = .001, p>.05. Similarly, the luxury condition did not have a main effect, F(1, 348) = 1.065, p>.05. However, the interaction was significant, F(1, 348) = 3.894, p < .05. Post-hoc testing using a Tukey correction found no significant differences. See Tables 2 and 3 for the omni-bus and post-hoc test results, respectively.

**Table 1:** ANOVA results for quantitative donation expectations.

Cases	Sum of Squares	df	Mean Square	F	p	η^2^
Moral Condition	97.761	1	97.761	0.114	0.735	< .001
Luxury Condition	239.413	1	239.413	0.280	0.597	< .001
Moral Condition * Luxury Condition	69.968	1	69.958	0.082	0.775	< .001
Residuals	297133.012	348	853.830			

**Table 2:** ANOVA results for qualitative donation expectations.

Cases	Sum of Squares	df	Mean Square	F	p	η^2^
Moral Condition	0.013	1	0.013	0.008	0.930	< .001
Luxury Condition	1.732	1	1.732	1.065	0.303	.003
Moral Condition * Luxury Condition	6.332	1	6.332	3.894	0.049	.011
Residuals	565.909	348	1.626			

**Table 3:** Post-hoc results for the interaction between the moral and luxury conditions.

Cases		Mean Difference	SE	t	p_tukey_
Immoral/Non-Luxury vs.	Moral/Non-Luxury	-0.283	0.207	-1.368	0.521
	Immoral/Luxury	-0.413	0.193	-2.136	0.144
	Moral/Luxury	-0.154	0.197	-0.782	0.862
Moral/Non-Luxury vs.	Immoral/Luxury	-0.130	0.192	-0.676	0.906
	Moral/Luxury	0.129	0.195	0.662	0.911
Immoral/Luxury vs.	Moral/Luxury	0.259	0.180	1.434	0.479

## Discussion

Study 3 continues to extend the results in the previous studies. Specifically, we find that the belief in reference-independence is stable even in light of a manipulation. For instance, it could be the case that a consumer holds objective standards, but when they are made aware that luxury firms contribute positively to society (i.e., are moral), they would lower these standards in comparison to standards for non-luxury firms. On the other hand, if consumers learn that luxury firms contribute negatively to society (i.e., are immoral), this knowledge may increase a firm’s expected donation standards as there would be a need for the firm to compensate for the societal ills caused. Our results suggest that neither is the case. When consumers are exposed to a written manipulation that emphasizes either the moral [immoral] aspects of the luxury industry, this manipulation does not alter a belief in reference-independence. Therefore, Study 3 uncovers the stability in objective standards for corporate giving.

## General discussion

Across four experimental studies, our research uncovers a unique aspect of consumer behavior—consumers hold objective, reference-independent beliefs about CSR expectations. While past research has uncovered a “pass the buck” effect in social interactions, whereby individuals apply subjective standards of generosity to wealthier others, we find that individuals do not “pass the buck” when making evaluations about companies. Instead, consumers apply more rigid and stable ethical expectations for firms. The research highlights an important boundary condition for the “pass the buck” effect, elaborating upon the way ethical beliefs may or may not vary based on the reference subject. Studies 1a and 1b answer the empirical question of whether consumers hold reference-dependent or reference-independent beliefs about CSR activities. Across two different product categories—automobiles and department stores—we find that consumers do not expect a higher standard of giving for the luxury firm. Study 2 continues to build upon this evidence by showcasing that when switching an individual’s perspective from a consumer to a manager, this objective ethical belief still remains. Study 3 explores how stable this belief is; even in light of stimuli meant to potentially alter the way consumers apply ethical standards of giving, we find that consumers’ beliefs in objective standards of giving prevails. Important to note, however, is the role that individual differences play. For example, consumers who scored higher on the spendthrift-tightwad scale expected larger amounts of donations from firms, regardless of if the firm sold luxury or non-luxury products. The same pattern of results held for materialistic consumers. Therefore, our results suggest that differential levels of donation expectations do exist—not for the evaluation of luxury or non-luxury firms, however—instead, these levels vary based upon the individual attributes of the consumer. In sum, while expectations for CSR activities is a reference-independent phenomenon, differences do exist among consumers regarding the level of donation expectation.

### Theoretical implications

Subjective ethical beliefs, or the idea that individuals make differential judgments about an ethical standard based on who or what the reference subject is, remains as an important topic across different disciplines. For example, in the field of moral philosophy, Feldman [53] writes how “[t]he crucial point so far is that objective justification in ethics is taken to be independent of beliefs or cognitive states of the agent. It in no way depends upon the agent’s perspective. Subjective justification, however, is relativized in one way or another to the cognitive state of the actor.” In medical ethics, some have pointed out that “objective scientific claims are often themselves subject to radical disagreement, even within the scientific community” [54]. Our research enters the discussion on the subjectivity of ethical standards by hypothesizing both normative and descriptive models of behavior [55]. A number of normative models of behavior exist; for instance, (1) individuals may hold all firms to the same standard, (2) those that make the most profit should donate the most, or (3) consumers may expect higher standards of giving from firms who contribute negatively to society. On the other hand, a descriptive model of judgment may include consumers holding higher standards for luxury firms given that they are associated with wealth and excess. Namely, while research has demonstrated that individuals follow a descriptive model of behavior when making judgments about donation standards [19], our research demonstrates that individuals follow a normative model of behavior: Regardless of the type of firm—and even in light of evidence suggesting moral [immoral] impacts—individuals hold steadfast beliefs about equal corporate contributions.

### Managerial implications

Managers for both luxury and non-luxury firms would benefit from understanding the way consumers hold reference-independent beliefs about CSR activities. Strategically, research has examined the way CSR can benefit a company’s bottom line. However, when stakeholders expect CSR activities, it is important for managers to know to what degree they should pursue these charitable activities. Given that corporations can pursue CSR as a method for demonstrating their commitment to societal well-being, the way in which consumers hold specific expectations becomes a relevant question. Our research findings suggest that managers for luxury firms need not pursue CSR above and beyond the activities employed by non-luxury firms. Instead, both luxury and non-luxury firms are held to a high standard of giving. In practice, for instance, when the luxury conglomerate LVMH decided to institute widespread CSR activities during the COVID-19 pandemic, consumers would also expect non-luxury firms to also commit to charitable causes; LVMH is not held to a higher standard than a non-luxury counterpart. These findings, especially in light of work by Sun, Bellezza [17], reveal interesting implications for luxury managers: (1) Consumers hold a belief that luxury goods will last longer (i.e., are more sustainable), and (2) there are actionable ways managers can nudge consumers in the direction of purchasing fewer but higher-end products; however, (3) emphasizing the sustainable features of luxury products does not reduce a consumer’s donation expectation.

Another managerial challenge for CSR activities includes the possibility for low levels of consumer awareness [56]. Although when consumers’ opinions about corporate donations are elicited, they hold consistent expectations across different companies, consumers may not be familiar with a company’s CSR agenda. However, research findings suggest a promising outcome for CSR activities: Once the consumer is made aware of a company’s corporate social contribution, for instance, consumers’ purchase intentions increase [57]. Some scholars have noted that a “pro-social” marketing communications and engagement approach can aid in increasing consumer awareness [56, 58]; yet, too much communication about CSR may not be always positive [15]. Finally, other research has demonstrated how consumers view core, central, and peripheral factors in the evaluation of a company’s CSR. In other words, there is “complexity of the evaluation process. This complexity may hinder even consumers with a positive attitude toward CSR to incorporate CSR into their decision-making process” [59]. At the same time, even when consumers do know about a luxury brand’s CSR activities, awareness may negatively affect consumers’ willingness to pay a premium price [60].

### Ideas for future research

Possibilities for future research are numerous. While we draw samples from a general population, future work that examines subjective ethical standards can examine cohort differences between (conspicuous and inconspicuous) luxury and non-luxury (e.g., mass prestige) consumers [33, 61]. For example, is there a difference between ethical donation standards when comparing luxury and non-luxury consumer samples? Past research has noted the divergent consumer behavior between luxury and non-luxury consumers [62]—in addition to divergence among luxury consumers. Given these differences, it would be interesting to understand how ethical beliefs may also be included. Furthermore, Berman, Bhattacharjee [19] hypothesize that “[a]lthough our studies focus on individual charitable donations, the effects we uncover likely apply towards resource contributions in social collectives and other organizational units.” We take up this call by examining how the same subjective donation standards that occur when individuals make social comparisons do not apply in the organizational context. However, future research can address *why* this may be the case. The present research uncovers this difference in reference-dependence, although we do not present the causal mechanisms that lead individuals to apply subjective standards in one case and objective standards in another. Finally, while our research finds that, from the perspective of the consumer, both luxury and non-luxury firms have similar obligation to engage in CSR activities, business ethicists can theorize how scholars can re-conceptualize the implicit contradiction between “luxury” and “sustainability.” Past research has noted this contradiction; future work can offer unifying theories that reconcile this “catch-22 of luxury” [63].

In addition to donations, consumers may expect luxury firms to contribute to diversity, equity, and inclusion initiatives. For instance, consumers may expect luxury firms to promote diversity in its corporate board composition, such as board gender diversity [64]. Extending this argument, a question can be asked if luxury (vs. non-luxury) firms have higher diversity expectations.

Finally, our results show correlational evidence for the role of spendthrift and materialism constructs for determining the level of corporate CSR expectations. Across a number of studies, we do find that materialists and spendthrifts hold higher donation standards than do non-materialists and tightwads, holding the type (luxury vs. non-luxury) of firm constant. However, the materialists and spendthrifts in our studies may have also differed on other variables of interest. Therefore, we suggest that future researchers interested in the role of the spendthrift and materialism constructs on CSR expectations experimentally manipulate these constructs through random assignment. We could imagine, for instance, a manipulation that involves watching different video stimuli that are associated with material (or non-material) themes [65]. Through this manipulation, researchers can more confidently narrow in on the causal impact of these constructs. In our studies, they are only treated as measures of individual difference. Furthermore, the mechanism(s) that lead(s) to higher donation standards for materialists (and spendthrifts) is (are) unclear from our studies, which serves as a limitation of the present research; however, we encourage future researchers to theorize and test potential mediators.

## Conclusion

Our research addresses an unanswered empirical question about the nature of the objectivity or subjectivity of ethical expectations for CSR activities. All studies presented in this research build a robust case that consumers hold a stable belief in the normative, reference-independent nature of corporate giving standards. In our view, the most salient limitation of our studies involves a discussion of statistical power. We conducted *a priori* power analyses for all of the studies based on a medium effect size, but some may argue that, perhaps, in order to uncover a statistically significant result (e.g., in the direction of reference-dependence), we needed more power (i.e., through a larger sample size). This may be the case if each study gave directional, but not significant, results in favor of luxury [non-luxury] firms having higher CSR expectations. However, across our studies, not only are the main effects not significant, but they also are not consistently in the same direction. We would also like to point to the effect sizes, which are virtually negligible. Therefore, we take such evidence to imply that consumers largely hold a reference-independent belief about CSR expectations.

Finally, our hope is that this research fosters interest in the emerging transformative luxury research movement. Luxury scholarship has the opportunity to move beyond the study of wealthy consumers who purchase expensive items; indeed, calls for transformative luxury research has noted that future work has the opportunity to understand the way luxury firms and products have the potential to contribute positively to society. Our research attempts to address this call by exploring the role that luxury firms (should) play in terms of engaging in charitable causes, which benefits both luxury and non-luxury consumers.
